# Training foundation doctors in mental health risk assessment as a tool in the fight against suicide

**DOI:** 10.1192/bjo.2021.150

**Published:** 2021-06-18

**Authors:** Jeorghino Lodge, Michael Obidoa

**Affiliations:** 1 Great Western Hospital NHS Foundation Trust; 2 Avon Wiltshire Mental Health Partnership

## Abstract

**Aims:**

To determine the perceptions of Junior Doctors on whether formal training in risk assessment could help to reduce the number of completed suicides following medical contact.

**Method:**

Foundation trainees within the Great Western Trust were surveyed using a questionnaire. For those trainees that were not present on the acute hospital site, the same questionnaire was distributed by the postgraduate medical team to all trainees using survey monkey. The survey was left open for four weeks. The total response rate was 57/88 foundation trainees. Simple statistical analysis of the data was performed and outlined below.

**Result:**

87% of all the trainees have never done a rotation in psychiatry. 51% of foundation doctors have had between 1-5 patients with suicidal behaviour or ideations admitted under the care of a medical team on which they were the junior doctor and up to 26% have admitted to encountering greater than 10 such patients. Only 37% of foundation trainees who have managed patients with suicidal behaviours admitted to having had any formal training in mental health risk assessment. Foundation trainees report being only somewhat confident in the identifying of factors that make a person high risk of completing suicide. 63% of all foundation trainees would refer any patient who expressed suicidal ideation for formal psychiatric assessment. Majority of the trainees were ‘not so confident’ in their ability to assess a patient's risk of suicide and in offering any help to mitigate this risk. None of the trainees have the intention to pursue psychiatry as a medical specialty and majority (60%) intend to pursue medical specialties. 56% of the trainees felt that training foundation doctors formally to assess patient mental health risk, could reduce the percentage of patients with completed suicide following being seen for non-psychiatric reason.

**Conclusion:**

The UK Foundation Program is a bridge that occupies that gap between undergraduate medical education and specialty training. It therefore an ideal opportunity for training clinicians in mental health risk assessment as one strategy to help reduce completed suicide following non-psychiatric health contact.

